# Pure Laparoscopic and Robot-Assisted Laparoscopic Reconstructive Surgery in Congenital Megaureter: A Single Institution Experience

**DOI:** 10.1371/journal.pone.0099777

**Published:** 2014-06-12

**Authors:** Weijun Fu, Xu Zhang, Xiaoyi Zhang, Peng Zhang, Jiangping Gao, Jun Dong, Guangfu Chen, Axiang Xu, Xin Ma, Hongzhao Li, Lixin Shi

**Affiliations:** 1 Department of Urology, PLA General Hospital/Medical school, Beijing, China; 2 Department of Urology, The Second Artillery General Hospital of PLA, Beijing, China; Centre for Inflammation Research, United Kingdom

## Abstract

To report our experience of pure laparoscopic and robot-assisted laparoscopic reconstructive surgery in congenital megaureter, seven patients (one bilateral) with symptomatic congenital megaureter underwent pure laparoscopic or robot-assisted laparoscopic surgery. The megaureter was exposed at the level of the blood vessel and was isolated to the bladder narrow area. Extreme ureter trim and submucosal tunnel encapsulation or papillary implantations and anti-reflux ureter bladder anastomosis were performed intraperitoneally by pure laparoscopic or robot-assisted laparoscopic surgery. The clinical data of seven patients after operation were analyzed, including the operation time, intraoperative complications, intraoperative bleeding volumes, postoperative complications, postoperative hospitalization time and pathological results. All of the patients were followed. The operation was successfully performed in seven patients. The mean operation times for pure laparoscopic surgery and robotic-assistant laparoscopic surgery were 175 (range: 150–220) and 187 (range: 170–205) min, respectively, and the mean operative blood loss volumes were 20 (range: 10–30) and 28.75 (range: 15–20) ml, respectively. There were no intraoperative complications. The postoperative drainage time was 5 (range: 4–6) and 5.75 (range: 5–6) d, respectively, and the indwelling catheter time was 6.33 (range: 4–8) d and 7 (range: 7–7) d, respectively. The postoperative hospitalization time was 7.67 (range: 7–8) d and 8 (range: 7–10) d, respectively. There was no obvious pain, no secondary bleeding and no urine leakage after the operation. Postoperative pathology reports revealed chronic urothelial mucosa inflammation. The follow-up results confirmed that all patients were relieved of their symptoms. Both pure laparoscopic and robot-assisted laparoscopic surgery using different anti-reflux ureter bladder anastomoses are safe and effective approaches in the minimally invasive treatment of congenital megaureter.

## Introduction

Congenital megaureter is a rare adult urinary tract anomaly that is due to distal ureteral muscle abnormalities, the loss of peristalsis from the vesicoureteral connection and vesicoureteral obstruction. Therefore, excision of the diseased segment plus with reimplantation of the distal ureter is often required.[1] Traditionally, open ureteral reparation and reimplantation is the gold standard therapy for primary symptomatic obstructive megaureter. Within the past decade, however, laparoscopic surgery has become popular in urology and has resulted in improved outcomes compared with open surgery, even in reconstruction procedures.[2] Today, the Da Vinci robot-assisted laparoscopic surgery platform (Intuitive Surgical, Sunnyvale, CA), which is characterized by stereoscopic vision and a flexible operating arm, provides us with the distinct advantages of minimally invasive reconstruction of the urinary tract, particularly in intracorporeal reparation and suturing. Particularly, for lower urinary tract reconstruction surgery, which is likely to be delayed by the narrow pelvic space, the robot-assisted laparoscopic surgery platform can be advantageous.[3] Here, we present our experience in the treatment of primary symptomatic obstructive megaureter by intracorporeal ureteral reparation, suturing and different anti-reflux methods using traditional pure laparoscopic surgery and robot-assisted laparoscopic surgery. To the best of our knowledge, this is the first time that ureteroneocystostomy has been reported to be performed by pure robotic repair followed by different anti-reflux strategies with a reasonable follow-up time.

## Materials and Methods

The study was reviewed and approved by an institutional review board (IRB) of the Chinese PLA Medical School. Written informed consent was obtained from all participants before the initiation of study procedures. The stored specimen and database analysis was approved by the IRB and ethics committee at Chinese PLA Medical School.

**Figure 1:**
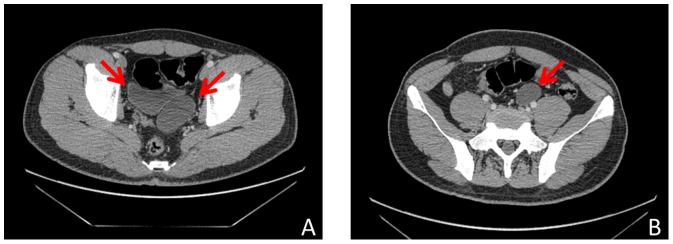
Preoperative CT scan of two patients. (A) The megaureter and bladder could be identified by CT scan. (B) The megaureter could be identified by CT scan.

We retrospectively reviewed seven patients (one of these patients exhibited bilateral megaureters) undergoing traditional pure laparoscopic surgery or robot-assisted laparoscopic surgery for primary symptomatic obstructive megaureter from December 2009 to August 2013 at PLA General Hospital (Beijing, China) (**Table 1**). There were two male patients and five female patients with a mean age of 28.14 years. All patients were evaluated by urinalysis, urine culture and assays of serum urea and creatinine levels as well as by imaging with ultrasonography, intravenous urography, computed tomography and emission computer tomography (ECT) renography. All surgeries were performed by experienced surgeons. (**Figure 1**)

**Table 1:** Patient general information.

Patient	Sex	Age	Magaureter Side	Method	Operating Time(min)	Bleeding (mL)	Follow-up Time (mo)	Result
1	F	24	Right	Laparoscopic	150	10	46	Relieved
2	M	20	Left	Laparoscopic	155	20	38	Relieved
3	F	37	Bilateral	Laparoscopic/Right	220	30	17	Relieved/Right
				Laparoscopic/Left				Relieved/Left
4	M	25	Left	Robot-assisted	190	20	3	Relieved
5	F	35	Left	Robot-assisted	170	15	57	Relieved
6	F	32	Left	Robot-assisted	205	30	4	Relieved
7	F	24	Left	Robot-assisted	185	50	3	Relieved

For traditional pure laparoscopic surgery, the patient was placed in a supine position. The primary 10-mm port for the camera was placed along the lower lip of the umbilicus in the midline, and 2 secondary ports (5 mm and 12 mm) were placed in the mid clavicular lines on either side 2 fingers lower than the camera port. An additional 5 port mm for the assistant was placed 2 cm above the iliac crest if necessary.

For robot-assisted laparoscopic surgery, a low dorsal lithotomy with a steep Trendelenburg position was used. Trocar placement was distributed to 5 points. A 12 mm port for the camera was placed three fingers superior to the umbilicus. Two 8-mm robotic ports for arm 1 and 2 were placed 8 cm lateral to umbilicus. A port for arm 3 was placed 8 cm contralaterally parallel to arm 1 or 2. An additional 12-mm port for the assistant was placed in the anterior axillary line 2 cm above the iliac crest on the ipsilateral surgical side of the ureter.

Monopolar scissors and bipolar grasping forceps were used to cut the Toldt line. The colon was reflected medially to expose the megaureter at the bifurcation of the common iliac vessels. The reproductive vessels were protected, and the megaureter was gently grasped with a latex band by robotic arm 3. The megaureter was isolated down to the distal ureteral stricture segment with attention to the preservation of the ureteral adventitia. The bladder was then filled with 150 ml normal saline and isolated fully along the Retzius space. The lateral top of the free bladder was fixed to the on psoas muscle by suturing, if necessary.

The ureter was partially amputated at the junction of the stricture and the dilated section of the ureter. To facilitated trimming and suturing, the ureter was drafted by a rubber belt or robot arm 3. The ureter was longitudinally sharply incised in a 3–4 cm extension over a 7-Fr plastic urethral catheter and, for heavy dilated in megaureter, intracorporeally tailored using a 4-zero absorbed polyglactin running suture. The ureteral stricture specimen was mobilized and excised for histopathological examination.

**Figure 2:**
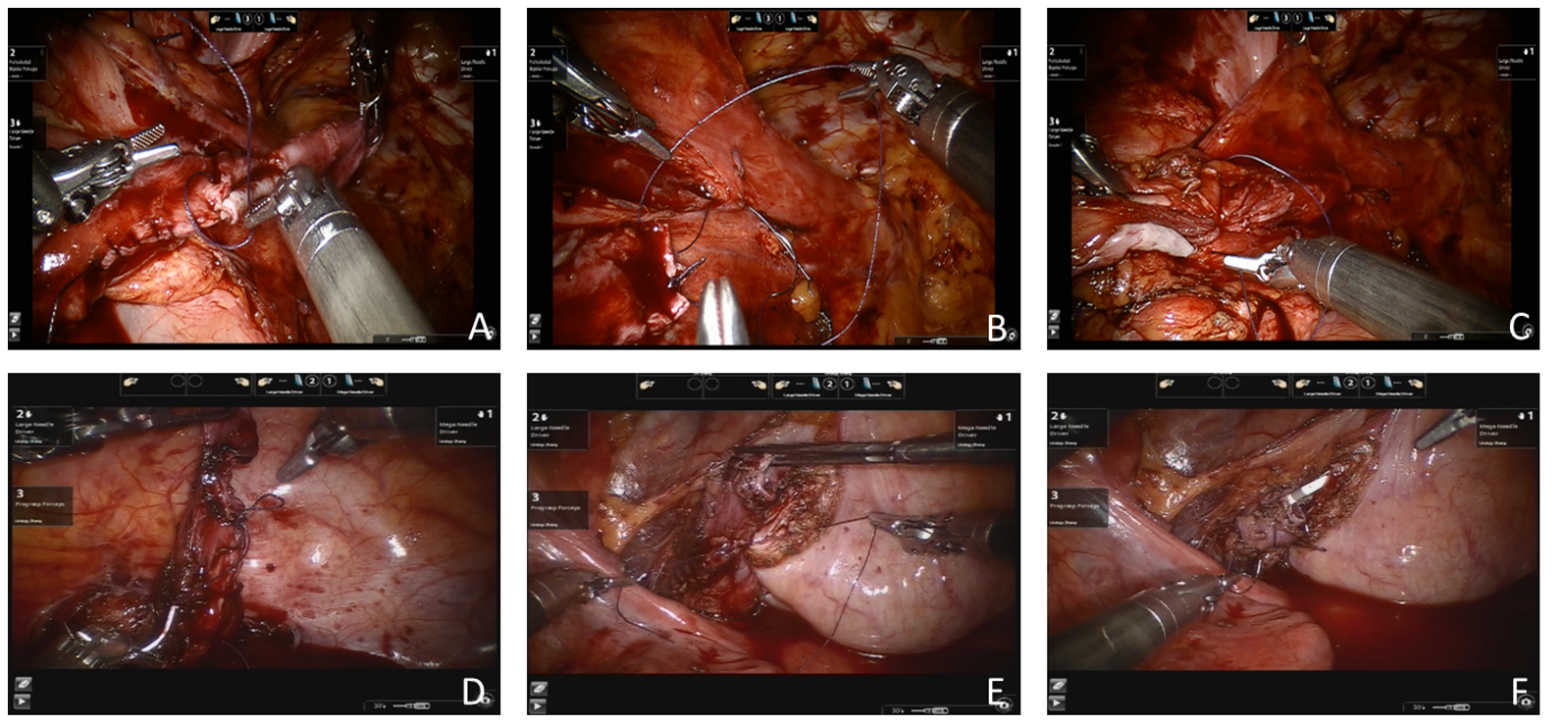
Robot-assisted laparoscopic surgery in congenital megaureter (A–C,submucosal tunnel reimplantation; D–E, direct ureteral nipple implantation). (A) The megaureter was intracorporeally tailored using a 4-zero absorbed polyglactin running suture. (B) The ureter was positioned through the submucosal tunnel and a mucosa-to-mucosa ureterovesical anastomosis was completed with 4-zero absorbed polyglactin interrupted sutures. (C) A running single layer suture with 2-zero polyglactin closed the bladder muscle. (D) The distal ureter was tailored and formed into a nipple. (E) The bladder was sutured full-thickness with the seromuscular layer to the ureter at a distance of 1.5 cm to the end at the 6 o'clock position to the anastomotic stoma. (F) After the fixation 4 interrupted sutures were made bilaterally to complete the anastomosis at the 12 o'clock position.

We applied two methods to achieve anti-reflux, submucosal tunnel reimplantation or ureteral nipple implantation according to the degree of dilated in megaureter of patients to avoid the post-operative stricture. For those seriously dilated megaureter we chose ureteral nipple implantation; however, for those slightly dilated megaureter we chose submucosal tunnel reimplantation. For submucosal tunnel reimplantation, the bladder was filled with normal saline and longitudinally opened in the posterolateral aspect to expose the mucosa of the bladder. A submucosal tunnel was created with blunt dissection at the lowest part of the bladder with a ratio of 5∶1 between the tunnel length and the diameter of the ureteral orifice. The ureter was positioned through the submucosal tunnel and a mucosa-to-mucosa ureterovesical anastomosis was completed with 4-zero absorbed polyglactin interrupted sutures. During this procedure, a 7Fr double J stent was passed into the ureter and advanced to the renal pelvis, and its distal end was fixed in the bladder. Subsequently, a running single layer suture with 2-zero polyglactin closed the bladder muscle. For this method, the ureter was covered with bladder detrusor muscle by suturing to form an anti-reflux submucosal tunnel (**Figure 2** **A–C**).

**Figure 3:**
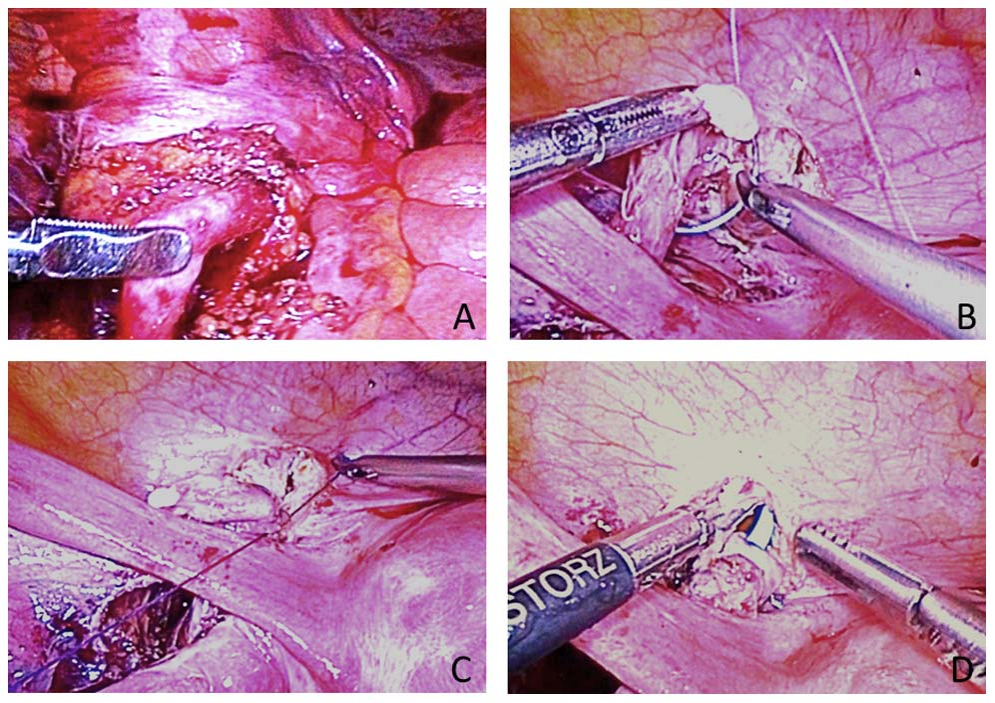
Pure laparoscopic surgery in congenital megaureter. (A) The megaureter was exposed at the bifurcation of the common iliac vessels. (B–C) The bladder was sutured full-thickness with the seromuscular layer to the ureter at a distance of 1.5 cm to the 6 o'clock position to the anastomotic stoma. (D) A 7-Fr double J stent was indwelled.

For ureteral nipple implantation, the distal ureter was tailored and formed into a nipple structure. Subsequently, the bladder detrusor and mucosa were dissected laterally. At the position 6 o'clock to the anastomotic stoma, the bladder was sutured full-thickness with the seromuscular layer to the ureter at a distance of 1.5 cm to the end. Subsequently, a 7-Fr double J stent was indwelled. After the fixation at 12 o'clock position of the anastomotic stoma, an additional 4 interrupted sutures were performed bilaterally to complete the anastomosis (**Figure 2** **D–F**, **Figure 3**).

A 14-Fr Nelaton drain was indwelled within the 5-mm port. Intravenous pyelography for the evaluation of residual obstruction and voiding cystourethrography for the evaluation of residual vesicoureteral reflux were performed postoperatively in all of the patients. Urinalysis, urine culture, assays of serum urea and creatinine levels and renal ultrasonography were also performed to evaluate renal function after the operation. The 7-Fr double J stent placed in operation was removed 4 weeks after operation by cystoscope.

## Results

**Figure 4:**
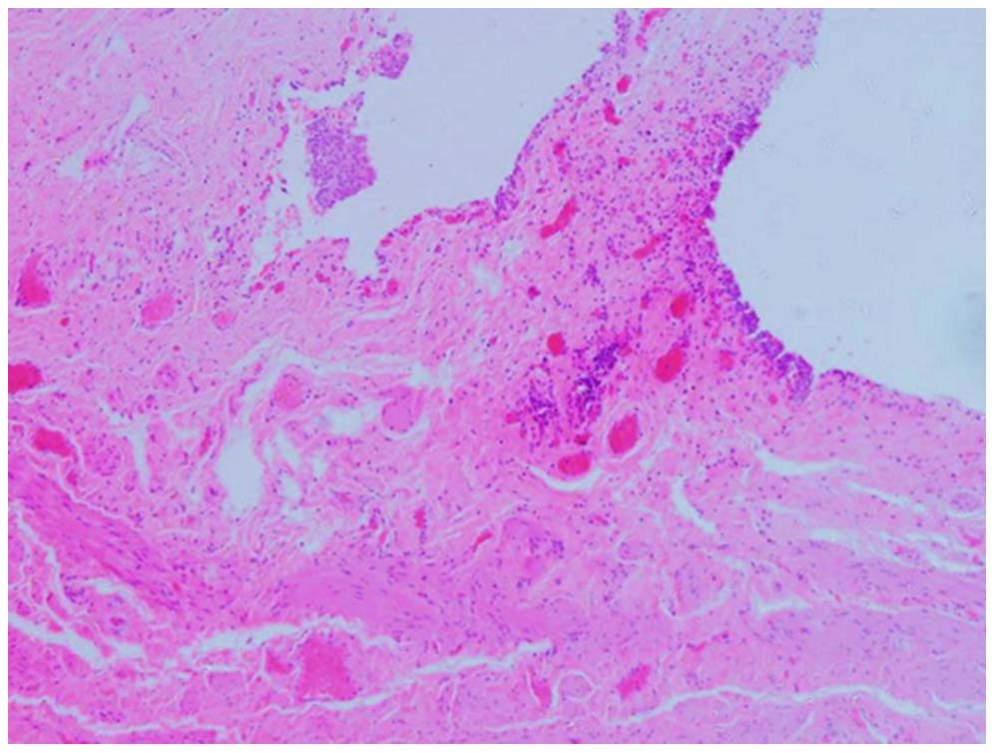
Histopathological reports for all the patients revealed chronic inflammation of the urothelial mucosa.

All of the operations were successfully completed. The mean operation time of pure laparoscopic surgery and robotic-assisted laparoscopic surgery were 175 (range: 150–220) min and 187 (range: 170–205) min, with an average intraoperative estimated blood loss of 20 (range: 10–30) ml and 28.75 (range: 15–20) ml, respectively. No major complications occurred during the two surgeries. Drainage times were 5 (range: 4–6) d and 5.75 (range: 5–6) d, respectively, and indwelling catheter times were 6.33 (range: 4–8) d and 7 (range: 7–7) d, respectively. The mean lengths of the postoperative hospital stay were 7.67 (range: 7–8) d and 8 (range: 7–10) d for the two groups, respectively. No significant postoperative pain, secondary bleeding, incontinence or other complications were observed after the operation. Histopathological reports for all of the patients revealed chronic inflammation of the urothelial mucosa (**Figure 4**). The double J ureteral stent was removed at 4 weeks after the operation.

**Figure 5:**
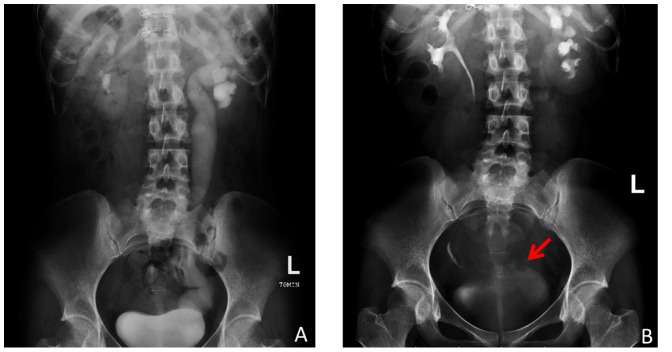
Preoperative and postoperative intravenous pyelography of one patient. (A) Preoperative intravenous pyelography of one megaureter patient. (B) Forty-months postoperative intravenous pyelography of one megaureter patient. The megaureter and hydronephrosis were relieved. The appearance of a filling defect due to the left nipple was identified.

The mean follow-up was 24 (range: 3–57) months. All patients experienced symptomatic relief. Follow-up ultrasonography and intravenous urography confirmed good drainage combined with a reduction of hydronephrosis. A statistically significant reduction was also achieved in the follow-up ureteral diameter compared with the preoperative ureteral diameter (**Figure 5**). The function of the salvaged kidney was preserved when compared with preoperative function. Nonobstructed clearance was also documented on diuretic renogram in all patients. The results retrograde cystogram also ensured that both of our two anti-reflux anastomses methods for reimplantation gained approving results. (Table 1).

## Discussion

Congenital megaureter is caused by a short or absent intravesical ureter, congenital para-ureteric diverticulum or other derangements of the vesico-ureteric junction (VUJ). According to the international classification, congenital megaureter can be classified as obstructed, refluxing or unobstructed and un-refluxing.[1] Congenital megaureter is most frequently observed in males of 30–40 years old. Most patients report lower back pain, and 1/3 present with stones. For clinical treatment, surgery of megaureter tailoring plus ureteroneocystostomy is recommended.[4]

Patients with congenital megaureter can undergo a variety of surgical treatment, including endoincision with stent indwelling, trimmed or not trimmed VUJ anastomosis or nephroureterectomy, on the basis of the ureter state and residual renal function. In situations of competent renal function, one commonly used surgical approach is to remove the non-functioning ureter followed by distal ureter bladder anastomosis. With traditional technical limitations, the open space of the narrow pelvis must be exposed during surgery, which requires for a large abdominal incision. This approach leads to increased complications and delayed postoperative recovery.[5]

To reduce complications and shorten recovery time, minimally invasive surgery, including endoscopic incision and laparoscopic ureteral reimplantation, has been proposed in recent years. Although transurethral endoscopic laser or electronic ureteral resection provided good short-term results, further observation of its long-term effects are needed. [6], [7] In recent years, laparoscopic ureteral reimplantation has been widely recommended in the treatment of congenital megaureter as a minimally invasive therapy. Pure laparoscopic reconstructive surgery requires extensive experience in laparoscopic surgery. Even for skilled surgeon, pure laparoscopic reconstructive surgery is a challenging technique.

The robot-assisted laparoscopic platform provides a new opportunity for complex urinary tract reconstruction surgery. The use of this approach in upper urinary tract reconstruction surgery has been widely reported and has proven to exhibit good long-term results.[8] Robot-assisted laparoscopic ureteral reimplantation, vesico-ureteric anastomosis, psoas muscle fixation and bladder valvuloplasty have also been reported, which have provided us with new approaches to the minimally invasive treatment of distal ureteral reconstruction.[9], [10] The robot-assisted surgery system has better dimensional vision and a more flexible operating arm and is therefore more suitable for narrow space reconstruction surgery.[11] In the lower urinary tract (lower ureter, the bladder and prostate) robot-assisted laparoscopic surgery, patients are placed in the low lithotomy position to facilitate the placement of the robot arm. Trocar placement for distal ureteral operations aims to provide the best operating space in the pelvic. During our surgery, robot arm 3 was used to hold the tissue dynamically. These alterations provide advantages by narrowing the necessary operating compared with pure laparoscopic surgeries.

In our single-institution study, we applied both pure laparoscopic and robot-assisted laparoscopic surgeries to patients with primary symptomatic obstructive megaureter. In both approaches, we tailored and sutured the megaureter intracorporeally to reduce the invasion of the ureter. In addition, we applied the vesico-ureteric anastomosis using two different methods, submucosal tunnel reimplantation or ureteral nipple implantation, to achieve an anti-reflux effect. To our knowledge, ureteral nipple implantation is not conventional according to the previous reports. [12], [13] Al-Shukri and Alwan first reported direct nipple ureteroneocystostomy in patients with ureteral stricture.[14] Here, we approved several cases of ureteral nipple implantation, including pure laparoscopic and robot-assisted laparoscopic surgery. In both pure laparoscopic and robot-assisted laparoscopic surgery, we perform direct nipple ureteroneocystostomy by suturing the bladder full-thickness with the seromuscular layer of the ureter at a distance of 1.5 cm to the end at the 6 and 12 o'clock positions of the anastomotic stoma. Subsequently, 4 additional interrupted sutures are made bilaterally to complete the anastomosis. During this procedure, the double J stent is placed. According to the postoperative examinations, the anti-reflux effect of this strategy has demonstrated that it is a simple, safe and feasible means of vesico-ureteric anastomosis. In particular, the ureteral nipple implantation shortens the operative time.

These groups of patients in our center have been treated with pure laparoscopic surgery or robotic-assisted laparoscopic surgery. Both approaches have reasonable and similar operating times and blood loss, but robotic ureteroneocystostomy is easier and takes less time than pure laparoscopic ureteroneocystostomy. However, robotic surgery is new to China, and with the accumulation of experiences, the operation time will be shortened further in the future. There were no major complications occurred during or after the operations. The drainage time, indwelling catheter times and postoperative hospital stays were similar for both the pure laparoscopic and robot-assisted laparoscopic surgeries. According to our latest follow-up, all of the patients were symptomatically recovered. Hydronephrosis in 8 megaureters of 7 patients was relieved as confirmed by postoperative examinations. The results of the follow-up have demonstrated that the salvaged kidneys well drained and resolved of hydronephrosis, and their functions were preserved when compared with the preoperative examinations.

## Conclusion

In summary, both pure laparoscopic and robot-assisted laparoscopic surgery for megaureter are technically feasible and safe. However, in our study, the number of cases is insufficient, and the follow-up time is short for some of the cases. More experience in robot-assisted laparoscopic surgery should be accumulated in future work. In the future, more clinical data are required to prove the convenience of robot-assisted laparoscopic surgery in complex genitourinary reconstructive operations.
